# 1-(3-Phenyl­isoquinolin-1-yl)hydrazine

**DOI:** 10.1107/S1600536808042062

**Published:** 2008-12-17

**Authors:** P. Manivel, Venkatesha R. Hathwar, P. Nithya, K. Prabakaran, F. Nawaz Khan

**Affiliations:** a Chemistry Division, School of Science and Humanities, VIT University, Vellore 632 014, Tamil Nadu, India; b Solid State and Structural Chemistry Unit, Indian Institute of Science, Bangalore 560 012, Karnataka, India

## Abstract

The title compound, C_15_H_13_N_3_, contains two independent mol­ecules in the asymmetric unit. The isoquinoline moiety and phenyl rings form dihedral angles of 4.38 (2) and 10.14 (3)° in the two independent mol­ecules. The crystal packing is stabilized by N—H⋯N mol­ecular dimers formed across a center of symmetry.

## Related literature

For general background to hydrazine compounds, see: Broadhurst *et al.* (2001 ▶); Behrens (1999 ▶); Broadhurst (1991 ▶); Chao *et al.* (1999 ▶); Kametani (1968 ▶). For related crystal structures, see: Yang *et al.* (2008 ▶); Choudhury & Guru Row (2006 ▶); Choudhury *et al.* (2002 ▶); Hathwar *et al.* (2008 ▶). For bond-length data, see: Allen *et al.* (1998 ▶). For hydrogen-bond motifs, see: Bernstein *et al.* (1995 ▶).
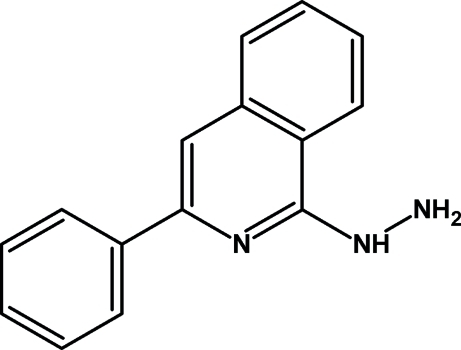


## Experimental

### 

#### Crystal data

C_15_H_13_N_3_
*M* *_r_* = 235.28
Triclinic, 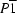

*a* = 6.672 (2) Å
*b* = 13.825 (4) Å
*c* = 14.934 (5) Å
α = 63.836 (5)°
β = 86.895 (6)°
γ = 82.106 (5)°
*V* = 1224.5 (7) Å^3^
*Z* = 4
Mo *K*α radiation
μ = 0.08 mm^−1^
*T* = 290 (2) K
0.15 × 0.12 × 0.05 mm

#### Data collection

Bruker SMART CCD area-detector diffractometer
Absorption correction: multi-scan (*SADABS*; Sheldrick, 1996 ▶) *T*_min_ = 0.953, *T*_max_ = 0.996
12381 measured reflections
4546 independent reflections
2926 reflections with *I* > 2σ(*I*)
*R*_int_ = 0.032

#### Refinement

*R*[*F* ^2^ > 2σ(*F* ^2^)] = 0.047
*wR*(*F* ^2^) = 0.116
*S* = 1.02
4546 reflections
429 parameters
All H-atom parameters refined
Δρ_max_ = 0.14 e Å^−3^
Δρ_min_ = −0.17 e Å^−3^

### 

Data collection: *SMART* (Bruker, 2004 ▶); cell refinement: *SAINT* (Bruker, 2004 ▶); data reduction: *SAINT*; program(s) used to solve structure: *SHELXS97* (Sheldrick, 2008 ▶); program(s) used to refine structure: *SHELXL97* (Sheldrick, 2008 ▶); molecular graphics: *ORTEP-3* (Farrugia,1999 ▶) and *CAMERON* (Watkin *et al.*, 1993 ▶); software used to prepare material for publication: *PLATON* (Spek, 2003 ▶).

## Supplementary Material

Crystal structure: contains datablocks global, I. DOI: 10.1107/S1600536808042062/cs2101sup1.cif

Structure factors: contains datablocks I. DOI: 10.1107/S1600536808042062/cs2101Isup2.hkl

Additional supplementary materials:  crystallographic information; 3D view; checkCIF report

## Figures and Tables

**Table 1:** Hydrogen-bond geometry (Å, °)

*D*—H⋯*A*	*D*—H	H⋯*A*	*D*⋯*A*	*D*—H⋯*A*
N2′—H2′*N*⋯N3′^i^	0.91 (2)	2.15 (2)	2.967 (2)	151 (2)
N2—H2*N*⋯N3^ii^	0.90 (2)	2.20 (2)	3.027 (2)	152 (2)
N3′—H3′*B*⋯N1′^iii^	0.89 (2)	2.24 (2)	3.119 (2)	169 (2)
N3—H3*A*⋯N1^iv^	0.92 (2)	2.26 (2)	3.170 (3)	168 (2)

Symmetry codes: (i) 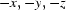; (ii) 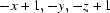; (iii) 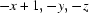; (iv) 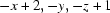.

**Fig. 1.**
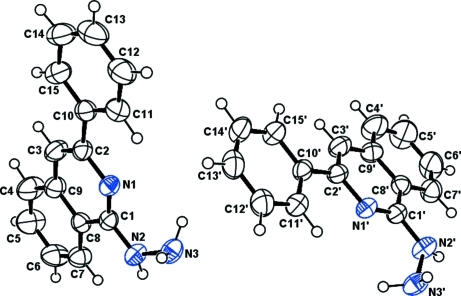
ORTEP diagram of the asymmetric unit of (I) with 50% probability displacement ellipsoids.

**Fig. 2.**
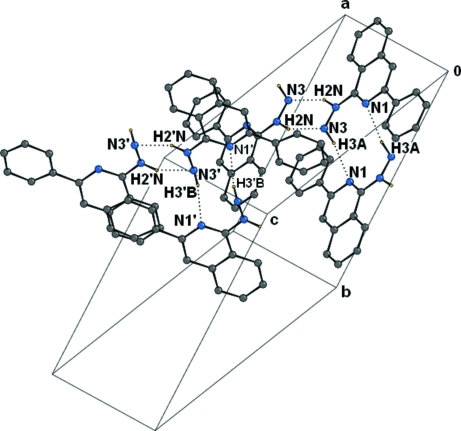
A packing excerpt from the crystal with dotted lines indicating intermolecular N—H···N hydrogen bonds. H atoms not involved in the interactions are omitted for clarity.

## supplementary crystallographic information

### Comment

The title compound belongs to the class isoquinolines. Isoquinolines and isoquinolinones are an integral part of many naturally occurring fused heterocycles and find applications in synthetic and pharmaceutical chemistry (Kametani *et al.*, 1968). Isoquinolinones and isoquinoline amines were reported as cancer chemotherapeutic agents (Behrens, 1999) whereas quinolyl and isoquinolyl derivatives have been reported as insecticidal compounds (Broadhurst, 1991). 3-Substituted isoquinolines are of potent use in medicine (Chao, *et al.*, 1999) and in general, hydrazine derivatives can be used as medicaments (Broadhurst *et al.*, 2001). Choudhury, *et al.* (2002, 2006) reported crystal structures of substituted isoquinolines while Hathwar, *et al.* (2008) reports the crystal structure of an isoquinolinyl diselenide.

The asymmetric unit of the crystal structure of the title compound contains two independent molecules (Fig. 1). The isoquinoline moiety and phenyl rings form dihedral angles of 4.38 (2) and 10.14 (3)° ,respectively, in the two independent molecules. All bond lengths and angles are normal (Allen *et al.*, 1998). The packing (Fig. 2) is consolidated by four N—H···N hydrogen bonds. All the four N—H···N hydrogen bonds generate dimers across centres of symmetry (Table 1) resulting in tight molecular packing in the crystal. The N2'-H2'N···N3' and the N2—H2N···N3 hydrogen bonds form a *R*^2^_2_(6) motif whereas the N3'-H3'B···N1' and the N3—H3A···N1 hydrogen bond dimers form a *R*^2^_2_(10) motif (Bernstein *et al.*, 1995) in the crystal (Fig. 2).

### Experimental

The solution of 1-chloro-3-phenylisoquioline in ethanol was treated with hydrazine hydrate and stirred at 323 K for 3hr. The product was filtered. The solid was washed with water and diethyl ether and dried under vacuum. Single crystals of the title compound were obtained via recrystalization from a dichloromethane solution.

### Refinement

All the H atoms in the title compound were located from difference electron density maps and refined isotropically resulting in C—H and N—H bond lenghts of 0.91 (4) - 1.02 (2)Å and 0.89 (2) - 0.97 (3)Å, respectively.

### Crystal data

**Table:**

C_15_H_13_N_3_	*Z* = 4
*M**_r_* = 235.28	*F*(000) = 496
Triclinic, *P*1	*D*_x_ = 1.276 Mg m^−^^3^
Hall symbol: -P 1	Mo *K*α radiation, λ = 0.71073 Å
*a* = 6.672 (2) Å	Cell parameters from 832 reflections
*b* = 13.825 (4) Å	θ = 1.7–25.3°
*c* = 14.934 (5) Å	µ = 0.08 mm^−^^1^
α = 63.836 (5)°	*T* = 290 K
β = 86.895 (6)°	Needle, colourless
γ = 82.106 (5)°	0.15 × 0.12 × 0.05 mm
*V* = 1224.5 (7) Å^3^	

### Data collection

**Table:**

Bruker SMART CCD area-detector diffractometer	4546 independent reflections
Radiation source: fine-focus sealed tube	2926 reflections with *I* > 2σ(*I*)
graphite	*R*_int_ = 0.032
φ and ω scans	θ_max_ = 25.5°, θ_min_ = 1.5°
Absorption correction: multi-scan (*SADABS*; Sheldrick, 1996)	*h* = −8→8
*T*_min_ = 0.953, *T*_max_ = 0.996	*k* = −16→16
12381 measured reflections	*l* = −18→18

### Refinement

**Table:**

Refinement on *F*^2^	Primary atom site location: structure-invariant direct methods
Least-squares matrix: full	Secondary atom site location: difference Fourier map
*R*[*F*^2^ > 2σ(*F*^2^)] = 0.047	Hydrogen site location: inferred from neighbouring sites
*wR*(*F*^2^) = 0.116	All H-atom parameters refined
*S* = 1.01	*w* = 1/[σ^2^(*F*_o_^2^) + (0.0567*P*)^2^] where *P* = (*F*_o_^2^ + 2*F*_c_^2^)/3
4546 reflections	(Δ/σ)_max_ < 0.001
429 parameters	Δρ_max_ = 0.14 e Å^−^^3^
0 restraints	Δρ_min_ = −0.17 e Å^−^^3^

### Fractional atomic coordinates and isotropic or equivalent isotropic displacement parameters (Å^2^)

**Table:**

	*x*	*y*	*z*	*U*_iso_*/*U*_eq_	
N1	0.91992 (19)	0.17183 (10)	0.48294 (10)	0.0386 (3)	
N2	0.6714 (2)	0.05976 (11)	0.52799 (11)	0.0433 (4)	
N3	0.7300 (2)	0.02645 (15)	0.45200 (14)	0.0471 (4)	
N1'	0.4205 (2)	0.13567 (10)	0.03997 (10)	0.0391 (3)	
N2'	0.1689 (2)	0.07962 (11)	−0.01722 (11)	0.0466 (4)	
N3'	0.2325 (2)	−0.03027 (12)	0.04958 (15)	0.0495 (4)	
C1	0.7552 (2)	0.14107 (12)	0.53468 (12)	0.0366 (4)	
C2	1.0076 (2)	0.25388 (13)	0.48758 (12)	0.0406 (4)	
C3	0.9290 (3)	0.30370 (15)	0.54472 (14)	0.0529 (5)	
C4	0.6656 (4)	0.32080 (18)	0.66201 (16)	0.0718 (7)	
C5	0.4979 (4)	0.28801 (19)	0.71627 (17)	0.0768 (7)	
C6	0.4084 (4)	0.20448 (17)	0.71345 (16)	0.0666 (6)	
C7	0.4885 (3)	0.15463 (16)	0.65585 (14)	0.0515 (5)	
C8	0.6624 (2)	0.18705 (12)	0.59912 (12)	0.0395 (4)	
C9	0.7543 (3)	0.27114 (14)	0.60246 (13)	0.0482 (5)	
C10	1.1910 (2)	0.28398 (13)	0.42535 (12)	0.0431 (4)	
C11	1.2553 (3)	0.23496 (16)	0.36341 (13)	0.0523 (5)	
C12	1.4255 (3)	0.26016 (18)	0.30579 (15)	0.0639 (6)	
C13	1.5362 (4)	0.33508 (19)	0.30874 (17)	0.0679 (6)	
C14	1.4761 (3)	0.38509 (18)	0.36893 (17)	0.0655 (6)	
C15	1.3048 (3)	0.36044 (16)	0.42676 (15)	0.0561 (5)	
C1'	0.2527 (2)	0.16171 (13)	−0.01268 (12)	0.0385 (4)	
C2'	0.5085 (2)	0.21556 (13)	0.04790 (12)	0.0406 (4)	
C3'	0.4252 (3)	0.32116 (15)	0.00313 (14)	0.0531 (5)	
C4'	0.1531 (4)	0.45988 (17)	−0.10239 (18)	0.0780 (7)	
C5'	−0.0202 (4)	0.48553 (19)	−0.15637 (19)	0.0876 (8)	
C6'	−0.1088 (4)	0.40594 (18)	−0.16610 (16)	0.0735 (7)	
C7'	−0.0217 (3)	0.30071 (16)	−0.12234 (14)	0.0547 (5)	
C8'	0.1569 (3)	0.27129 (13)	−0.06543 (12)	0.0411 (4)	
C9'	0.2471 (3)	0.35185 (14)	−0.05511 (13)	0.0502 (5)	
C10'	0.6945 (2)	0.17691 (14)	0.11012 (12)	0.0412 (4)	
C11'	0.7519 (3)	0.06689 (16)	0.16619 (14)	0.0525 (5)	
C12'	0.9251 (3)	0.02867 (18)	0.22499 (15)	0.0599 (5)	
C13'	1.0450 (3)	0.09997 (19)	0.22820 (15)	0.0587 (5)	
C14'	0.9911 (3)	0.2093 (2)	0.17264 (15)	0.0608 (6)	
C15'	0.8188 (3)	0.24744 (17)	0.11461 (15)	0.0532 (5)	
H2N	0.542 (3)	0.0483 (14)	0.5454 (13)	0.060 (6)*	
H3	0.992 (3)	0.3609 (14)	0.5471 (12)	0.056 (5)*	
H3A	0.843 (3)	−0.0248 (16)	0.4726 (14)	0.075 (7)*	
H3B	0.761 (3)	0.0880 (17)	0.3956 (15)	0.078 (7)*	
H4	0.733 (3)	0.3797 (16)	0.6607 (14)	0.082 (7)*	
H5	0.438 (3)	0.3227 (17)	0.7544 (16)	0.094 (7)*	
H2'N	0.038 (3)	0.0910 (13)	−0.0365 (12)	0.053 (5)*	
H6	0.286 (3)	0.1827 (16)	0.7500 (15)	0.084 (7)*	
H3'A	0.269 (3)	−0.0320 (14)	0.1126 (14)	0.065 (6)*	
H3'B	0.341 (3)	−0.0536 (16)	0.0238 (15)	0.076 (7)*	
H7	0.423 (3)	0.1003 (14)	0.6516 (12)	0.055 (5)*	
H11	1.172 (3)	0.1806 (14)	0.3645 (13)	0.062 (5)*	
H12	1.470 (3)	0.2194 (16)	0.2639 (15)	0.090 (7)*	
H13	1.658 (3)	0.3473 (17)	0.2734 (16)	0.095 (8)*	
H14	1.549 (3)	0.4356 (15)	0.3753 (13)	0.069 (6)*	
H15	1.263 (3)	0.3971 (14)	0.4688 (13)	0.059 (6)*	
H3'	0.492 (3)	0.3731 (15)	0.0110 (13)	0.064 (6)*	
H4'	0.219 (3)	0.5136 (17)	−0.0959 (14)	0.082 (7)*	
H5'	−0.082 (4)	0.5558 (19)	−0.1851 (17)	0.102 (8)*	
H6'	−0.234 (3)	0.4247 (16)	−0.2025 (15)	0.080 (7)*	
H7'	−0.081 (3)	0.2459 (15)	−0.1303 (13)	0.065 (6)*	
H11'	0.661 (3)	0.0177 (14)	0.1642 (12)	0.057 (5)*	
H12'	0.964 (3)	−0.0520 (18)	0.2615 (15)	0.092 (7)*	
H13'	1.164 (3)	0.0723 (15)	0.2692 (14)	0.070 (6)*	
H14'	1.073 (3)	0.2590 (16)	0.1730 (14)	0.078 (6)*	
H15'	0.786 (3)	0.3249 (15)	0.0727 (14)	0.069 (6)*	

### Atomic displacement parameters (Å^2^)

**Table:**

	*U*^11^	*U*^22^	*U*^33^	*U*^12^	*U*^13^	*U*^23^
N1	0.0333 (8)	0.0399 (8)	0.0440 (8)	−0.0050 (6)	−0.0015 (6)	−0.0194 (7)
N2	0.0385 (9)	0.0477 (9)	0.0538 (9)	−0.0105 (7)	0.0057 (7)	−0.0306 (8)
N3	0.0416 (9)	0.0510 (10)	0.0609 (11)	−0.0049 (8)	0.0013 (8)	−0.0358 (9)
N1'	0.0349 (8)	0.0393 (8)	0.0494 (8)	−0.0077 (6)	0.0027 (7)	−0.0245 (7)
N2'	0.0365 (9)	0.0386 (9)	0.0688 (11)	−0.0028 (7)	−0.0065 (8)	−0.0271 (8)
N3'	0.0420 (10)	0.0382 (9)	0.0720 (12)	−0.0014 (7)	−0.0050 (9)	−0.0281 (9)
C1	0.0341 (9)	0.0347 (9)	0.0398 (9)	−0.0008 (7)	−0.0057 (8)	−0.0155 (8)
C2	0.0388 (10)	0.0401 (10)	0.0416 (10)	−0.0052 (8)	−0.0054 (8)	−0.0161 (8)
C3	0.0572 (12)	0.0539 (12)	0.0606 (12)	−0.0214 (10)	0.0080 (10)	−0.0337 (10)
C4	0.0929 (18)	0.0715 (15)	0.0772 (15)	−0.0321 (13)	0.0289 (13)	−0.0532 (13)
C5	0.0979 (19)	0.0770 (16)	0.0769 (16)	−0.0248 (14)	0.0364 (14)	−0.0532 (14)
C6	0.0735 (15)	0.0659 (14)	0.0659 (14)	−0.0203 (12)	0.0312 (12)	−0.0341 (12)
C7	0.0539 (12)	0.0495 (11)	0.0541 (12)	−0.0111 (10)	0.0095 (9)	−0.0250 (10)
C8	0.0416 (10)	0.0374 (9)	0.0368 (9)	−0.0013 (8)	−0.0019 (8)	−0.0145 (8)
C9	0.0549 (12)	0.0462 (11)	0.0496 (11)	−0.0100 (9)	0.0041 (9)	−0.0259 (9)
C10	0.0393 (10)	0.0408 (10)	0.0415 (10)	−0.0048 (8)	−0.0055 (8)	−0.0106 (8)
C11	0.0525 (12)	0.0569 (12)	0.0458 (11)	−0.0127 (10)	0.0048 (9)	−0.0198 (10)
C12	0.0592 (14)	0.0723 (15)	0.0525 (13)	−0.0113 (11)	0.0097 (10)	−0.0205 (12)
C13	0.0505 (14)	0.0723 (15)	0.0578 (14)	−0.0092 (12)	0.0081 (11)	−0.0081 (12)
C14	0.0508 (13)	0.0575 (13)	0.0733 (15)	−0.0191 (11)	−0.0029 (12)	−0.0115 (12)
C15	0.0510 (12)	0.0508 (12)	0.0652 (14)	−0.0133 (10)	0.0005 (10)	−0.0223 (11)
C1'	0.0354 (10)	0.0413 (10)	0.0454 (10)	−0.0068 (8)	0.0065 (8)	−0.0252 (8)
C2'	0.0408 (10)	0.0411 (10)	0.0447 (10)	−0.0115 (8)	0.0077 (8)	−0.0223 (8)
C3'	0.0611 (13)	0.0420 (11)	0.0600 (12)	−0.0146 (10)	−0.0037 (10)	−0.0230 (10)
C4'	0.1021 (19)	0.0388 (12)	0.0861 (17)	−0.0059 (12)	−0.0263 (15)	−0.0189 (12)
C5'	0.115 (2)	0.0430 (14)	0.0886 (18)	0.0131 (14)	−0.0384 (16)	−0.0160 (13)
C6'	0.0877 (18)	0.0566 (14)	0.0677 (15)	0.0104 (13)	−0.0303 (13)	−0.0215 (12)
C7'	0.0602 (13)	0.0500 (12)	0.0537 (12)	−0.0013 (10)	−0.0095 (10)	−0.0231 (10)
C8'	0.0450 (10)	0.0400 (10)	0.0381 (10)	−0.0051 (8)	0.0030 (8)	−0.0174 (8)
C9'	0.0620 (13)	0.0359 (10)	0.0505 (11)	−0.0080 (9)	−0.0019 (10)	−0.0161 (9)
C10'	0.0406 (10)	0.0492 (11)	0.0424 (10)	−0.0116 (8)	0.0061 (8)	−0.0269 (9)
C11'	0.0573 (13)	0.0514 (12)	0.0513 (12)	−0.0153 (10)	−0.0051 (10)	−0.0217 (10)
C12'	0.0654 (14)	0.0609 (14)	0.0523 (12)	−0.0064 (11)	−0.0107 (10)	−0.0232 (11)
C13'	0.0533 (13)	0.0777 (16)	0.0502 (12)	−0.0078 (12)	−0.0060 (10)	−0.0322 (12)
C14'	0.0571 (13)	0.0779 (16)	0.0612 (13)	−0.0257 (12)	−0.0016 (11)	−0.0380 (13)
C15'	0.0556 (12)	0.0543 (13)	0.0575 (13)	−0.0162 (10)	−0.0002 (10)	−0.0288 (11)

### Geometric parameters (Å, °)

**Table:**

N1—C1	1.3163 (19)	C12—C13	1.369 (3)
N1—C2	1.3748 (19)	C12—H12	1.02 (2)
N2—C1	1.3652 (19)	C13—C14	1.371 (3)
N2—N3	1.420 (2)	C13—H13	0.94 (2)
N2—H2N	0.904 (18)	C14—C15	1.381 (3)
N3—H3A	0.920 (19)	C14—H14	0.94 (2)
N3—H3B	0.93 (2)	C15—H15	0.976 (17)
N1'—C1'	1.317 (2)	C1'—C8'	1.435 (2)
N1'—C2'	1.3712 (19)	C2'—C3'	1.358 (2)
N2'—C1'	1.360 (2)	C2'—C10'	1.481 (2)
N2'—N3'	1.420 (2)	C3'—C9'	1.413 (3)
N2'—H2'N	0.903 (18)	C3'—H3'	0.946 (18)
N3'—H3'A	0.976 (19)	C4'—C5'	1.360 (3)
N3'—H3'B	0.89 (2)	C4'—C9'	1.408 (3)
C1—C8	1.442 (2)	C4'—H4'	0.96 (2)
C2—C3	1.359 (2)	C5'—C6'	1.382 (3)
C2—C10	1.487 (2)	C5'—H5'	0.92 (2)
C3—C9	1.414 (2)	C6'—C7'	1.361 (3)
C3—H3	0.959 (17)	C6'—H6'	0.96 (2)
C4—C5	1.353 (3)	C7'—C8'	1.407 (2)
C4—C9	1.408 (2)	C7'—H7'	0.955 (18)
C4—H4	0.98 (2)	C8'—C9'	1.404 (2)
C5—C6	1.388 (3)	C10'—C11'	1.383 (2)
C5—H5	0.94 (2)	C10'—C15'	1.389 (2)
C6—C7	1.368 (3)	C11'—C12'	1.386 (3)
C6—H6	0.96 (2)	C11'—H11'	0.983 (17)
C7—C8	1.402 (2)	C12'—C13'	1.370 (3)
C7—H7	0.945 (17)	C12'—H12'	1.00 (2)
C8—C9	1.407 (2)	C13'—C14'	1.370 (3)
C10—C11	1.389 (2)	C13'—H13'	0.95 (2)
C10—C15	1.391 (2)	C14'—C15'	1.374 (3)
C11—C12	1.375 (3)	C14'—H14'	0.94 (2)
C11—H11	0.988 (18)	C15'—H15'	0.972 (18)
			
C1—N1—C2	119.22 (14)	C14—C13—H13	120.8 (13)
C1—N2—N3	121.14 (14)	C13—C14—C15	120.6 (2)
C1—N2—H2N	122.1 (11)	C13—C14—H14	123.3 (12)
N3—N2—H2N	109.5 (11)	C15—C14—H14	116.1 (12)
N2—N3—H3A	109.6 (12)	C14—C15—C10	120.6 (2)
N2—N3—H3B	107.2 (12)	C14—C15—H15	119.7 (11)
H3A—N3—H3B	109.9 (18)	C10—C15—H15	119.8 (11)
C1'—N1'—C2'	119.52 (14)	N1'—C1'—N2'	117.49 (15)
C1'—N2'—N3'	120.66 (15)	N1'—C1'—C8'	123.38 (14)
C1'—N2'—H2'N	119.4 (11)	N2'—C1'—C8'	119.13 (15)
N3'—N2'—H2'N	112.8 (11)	C3'—C2'—N1'	121.27 (16)
N2'—N3'—H3'A	107.7 (11)	C3'—C2'—C10'	123.79 (16)
N2'—N3'—H3'B	108.0 (13)	N1'—C2'—C10'	114.93 (15)
H3'A—N3'—H3'B	109.2 (17)	C2'—C3'—C9'	120.53 (17)
N1—C1—N2	117.71 (15)	C2'—C3'—H3'	117.9 (11)
N1—C1—C8	123.47 (14)	C9'—C3'—H3'	121.5 (11)
N2—C1—C8	118.81 (15)	C5'—C4'—C9'	121.0 (2)
C3—C2—N1	121.64 (16)	C5'—C4'—H4'	122.4 (12)
C3—C2—C10	123.14 (16)	C9'—C4'—H4'	116.6 (12)
N1—C2—C10	115.22 (15)	C4'—C5'—C6'	120.7 (2)
C2—C3—C9	120.43 (17)	C4'—C5'—H5'	120.1 (15)
C2—C3—H3	120.7 (10)	C6'—C5'—H5'	119.2 (15)
C9—C3—H3	118.9 (10)	C7'—C6'—C5'	120.1 (2)
C5—C4—C9	121.3 (2)	C7'—C6'—H6'	119.9 (12)
C5—C4—H4	123.4 (12)	C5'—C6'—H6'	120.0 (12)
C9—C4—H4	115.3 (12)	C6'—C7'—C8'	120.6 (2)
C4—C5—C6	120.2 (2)	C6'—C7'—H7'	120.2 (11)
C4—C5—H5	120.7 (14)	C8'—C7'—H7'	119.2 (11)
C6—C5—H5	119.0 (14)	C9'—C8'—C7'	119.44 (17)
C7—C6—C5	120.5 (2)	C9'—C8'—C1'	116.33 (15)
C7—C6—H6	119.1 (12)	C7'—C8'—C1'	124.19 (16)
C5—C6—H6	120.4 (12)	C8'—C9'—C4'	118.12 (19)
C6—C7—C8	120.42 (19)	C8'—C9'—C3'	118.93 (16)
C6—C7—H7	120.1 (10)	C4'—C9'—C3'	122.94 (18)
C8—C7—H7	119.4 (10)	C11'—C10'—C15'	117.18 (17)
C7—C8—C9	119.24 (16)	C11'—C10'—C2'	120.30 (16)
C7—C8—C1	124.44 (16)	C15'—C10'—C2'	122.51 (17)
C9—C8—C1	116.31 (15)	C10'—C11'—C12'	121.42 (18)
C8—C9—C4	118.37 (18)	C10'—C11'—H11'	116.7 (10)
C8—C9—C3	118.91 (16)	C12'—C11'—H11'	121.9 (10)
C4—C9—C3	122.71 (18)	C13'—C12'—C11'	120.2 (2)
C11—C10—C15	117.55 (18)	C13'—C12'—H12'	121.5 (12)
C11—C10—C2	120.17 (16)	C11'—C12'—H12'	118.2 (12)
C15—C10—C2	122.27 (17)	C12'—C13'—C14'	119.1 (2)
C12—C11—C10	121.6 (2)	C12'—C13'—H13'	119.1 (12)
C12—C11—H11	122.8 (10)	C14'—C13'—H13'	121.8 (12)
C10—C11—H11	115.6 (10)	C13'—C14'—C15'	120.8 (2)
C13—C12—C11	120.0 (2)	C13'—C14'—H14'	120.1 (12)
C13—C12—H12	121.3 (12)	C15'—C14'—H14'	119.1 (12)
C11—C12—H12	118.6 (12)	C14'—C15'—C10'	121.3 (2)
C12—C13—C14	119.8 (2)	C14'—C15'—H15'	119.9 (11)
C12—C13—H13	119.3 (13)	C10'—C15'—H15'	118.8 (11)
			
C2—N1—C1—N2	179.67 (14)	C2'—N1'—C1'—N2'	−179.00 (14)
C2—N1—C1—C8	−1.7 (2)	C2'—N1'—C1'—C8'	1.6 (2)
N3—N2—C1—N1	−14.0 (2)	N3'—N2'—C1'—N1'	14.0 (2)
N3—N2—C1—C8	167.31 (15)	N3'—N2'—C1'—C8'	−166.61 (15)
C1—N1—C2—C3	0.4 (2)	C1'—N1'—C2'—C3'	0.5 (2)
C1—N1—C2—C10	−179.08 (13)	C1'—N1'—C2'—C10'	179.06 (14)
N1—C2—C3—C9	0.7 (3)	N1'—C2'—C3'—C9'	−1.5 (3)
C10—C2—C3—C9	−179.86 (15)	C10'—C2'—C3'—C9'	−179.91 (15)
C9—C4—C5—C6	−0.6 (4)	C9'—C4'—C5'—C6'	0.1 (4)
C4—C5—C6—C7	−0.1 (4)	C4'—C5'—C6'—C7'	−0.7 (4)
C5—C6—C7—C8	0.2 (3)	C5'—C6'—C7'—C8'	1.1 (3)
C6—C7—C8—C9	0.3 (3)	C6'—C7'—C8'—C9'	−0.9 (3)
C6—C7—C8—C1	−178.62 (17)	C6'—C7'—C8'—C1'	176.68 (18)
N1—C1—C8—C7	−179.17 (15)	N1'—C1'—C8'—C9'	−2.6 (2)
N2—C1—C8—C7	−0.6 (2)	N2'—C1'—C8'—C9'	177.99 (15)
N1—C1—C8—C9	1.9 (2)	N1'—C1'—C8'—C7'	179.72 (16)
N2—C1—C8—C9	−179.52 (14)	N2'—C1'—C8'—C7'	0.3 (2)
C7—C8—C9—C4	−0.9 (3)	C7'—C8'—C9'—C4'	0.3 (3)
C1—C8—C9—C4	178.12 (17)	C1'—C8'—C9'—C4'	−177.49 (17)
C7—C8—C9—C3	−179.72 (16)	C7'—C8'—C9'—C3'	179.33 (17)
C1—C8—C9—C3	−0.7 (2)	C1'—C8'—C9'—C3'	1.6 (2)
C5—C4—C9—C8	1.0 (3)	C5'—C4'—C9'—C8'	0.1 (3)
C5—C4—C9—C3	179.8 (2)	C5'—C4'—C9'—C3'	−178.9 (2)
C2—C3—C9—C8	−0.5 (3)	C2'—C3'—C9'—C8'	0.4 (3)
C2—C3—C9—C4	−179.28 (18)	C2'—C3'—C9'—C4'	179.37 (19)
C3—C2—C10—C11	−175.55 (17)	C3'—C2'—C10'—C11'	169.50 (17)
N1—C2—C10—C11	3.9 (2)	N1'—C2'—C10'—C11'	−9.0 (2)
C3—C2—C10—C15	4.8 (3)	C3'—C2'—C10'—C15'	−11.0 (3)
N1—C2—C10—C15	−175.73 (15)	N1'—C2'—C10'—C15'	170.47 (15)
C15—C10—C11—C12	0.4 (3)	C15'—C10'—C11'—C12'	0.5 (3)
C2—C10—C11—C12	−179.27 (16)	C2'—C10'—C11'—C12'	180.00 (16)
C10—C11—C12—C13	0.1 (3)	C10'—C11'—C12'—C13'	−0.4 (3)
C11—C12—C13—C14	−0.3 (3)	C11'—C12'—C13'—C14'	0.1 (3)
C12—C13—C14—C15	0.1 (3)	C12'—C13'—C14'—C15'	0.3 (3)
C13—C14—C15—C10	0.4 (3)	C13'—C14'—C15'—C10'	−0.2 (3)
C11—C10—C15—C14	−0.6 (3)	C11'—C10'—C15'—C14'	−0.2 (3)
C2—C10—C15—C14	179.05 (16)	C2'—C10'—C15'—C14'	−179.68 (16)

### Hydrogen-bond geometry (Å, °)

**Table:**

*D*—H···*A*	*D*—H	H···*A*	*D*···*A*	*D*—H···*A*
N2'—H2'N···N3'^i^	0.91 (2)	2.15 (2)	2.967 (2)	151 (2)
N2—H2N···N3^ii^	0.90 (2)	2.20 (2)	3.027 (2)	152 (2)
N3'—H3'B···N1'^iii^	0.89 (2)	2.24 (2)	3.119 (2)	169 (2)
N3—H3A···N1^iv^	0.92 (2)	2.26 (2)	3.170 (3)	168 (2)

Symmetry codes: (i) −*x*, −*y*, −*z*; (ii) −*x*+1, −*y*, −*z*+1; (iii) −*x*+1, −*y*, −*z*; (iv) −*x*+2, −*y*, −*z*+1.
